# Circulating amyloid beta 1‐40 peptide as an associate of renal function decline

**DOI:** 10.1111/eci.70006

**Published:** 2025-02-24

**Authors:** Georgios Mavraganis, Georgios Georgiopoulos, Georgios Zervas, Evmorfia Aivalioti, Dimitrios Delialis, Ioannis Petropoulos, Nikolaos Rachiotis, Christina Konstantaki, Chrysoula Moustou, Maria‐Aggeliki Dimopoulou, Marco Sachse, Simon Tual‐Chalot, Kateryna Sopova, Erasmia Psimmenou, Konstantinos Stellos, Kimon Stamatelopoulos

**Affiliations:** ^1^ Department of Clinical Therapeutics, Alexandra Hospital National and Kapodistrian University of Athens Medical School Athens Greece; ^2^ Department of Physiology, School of Medicine University of Patras Patras Greece; ^3^ Department of Cardiovascular Surgery, University Heart and Vascular Centre University Medical Centre Hamburg‐Eppendorf Hamburg Germany; ^4^ Department of Cardiovascular Research, Medical Faculty Mannheim Heidelberg University Mannheim Germany; ^5^ Biosciences Institute, Vascular Biology and Medicine Theme, Faculty of Medical Sciences Newcastle University Newcastle upon Tyne UK; ^6^ Department of Medicine, University Medical Centre Mannheim Heidelberg University Mannheim Germany; ^7^ German Centre for Cardiovascular Research (DZHK), Partner Site Heidelberg/Mannheim Mannheim Germany; ^8^ Helmholtz Institute for Translational AngioCardioScience (HI‐TAC) Mannheim Germany

**Keywords:** all‐cause mortality, amyloid Αβ1‐40, glomerular filtration rate, kidney function, mediation analysis

## Abstract

**Background:**

Recent evidence suggests that Alzheimer's amyloid‐beta (1‐40) (Αβ1‐40), an emerging biomarker of cardiovascular disease, may be involved in the heart‐brain‐renal axis. We aimed to comprehensively explore the association between circulating Aβ1‐40 levels and renal function and its clinical relevance.

**Methods:**

Consecutively recruited subjects in the Athens Angiometabolic Registry with measured Aβ1‐40 plasma levels (*n* = 811) were analysed. Αβ1‐40 was measured by enzyme‐linked immunosorbent assay and glomerular filtration rate (GFR) was calculated using the abbreviated four‐variable Modification of Diet in Renal Disease (MDRD) formula. All‐cause mortality was the main clinical endpoint across a median follow‐up of 47 months.

**Results:**

Cross‐sectionally, a bidirectional association between Αβ1‐40 [adjusted odds ratio (adjOR) = 3.67 for highest tertile of Αβ1‐40 and chronic kidney disease (CKD) stage ≥3, *p* < .001] and CKD stage ≥3 (adjOR = 3.52 for association with highest Aβ1‐40 tertile, *p* < .001) was observed. Longitudinally, increased Αβ1‐40 at baseline was associated with decline in renal function at follow‐up (adjOR for CKD stage ≥3 = 2.26, *p* = .033). Similarly, longitudinal changes in Aβ1‐40 were inversely associated with changes in GFR (OR = .77 per 1 SD increase in Aβ1‐40, *p* = .006). Aβ1‐40 was associated with all‐cause mortality, independently of traditional risk factors (hazard ratio = 1.20 per 1 SD increase in Aβ1‐40, *p* = .016). An indirect effect of GFR on the association between Aβ1‐40 and mortality (*p* < .05) with an estimated indirect‐to‐total effect ratio of .334, but not of Αβ1‐40 on GFR with mortality, was observed.

**Conclusions:**

In a population with a wide range of GFR, we found a bidirectional association between Αβ1‐40 levels and renal function. The association of Αβ1‐40 with all‐cause mortality was partly mediated by lower GFR.

## Graphical abstract

In this cohort study, we explored the association between amyloid‐beta 1‐40 (Aβ1‐40) plasma levels and kidney function and its clinical implications in 811 consecutive individuals with variable cardiovascular risk. Increased Aβ1‐40 was bidirectionally and consistently associated with worse kidney function at two different time points. Higher Aβ1‐40 predicted glomerular filtration rate (GFR) decline over time. Finally, we observed an independent association of increased Aβ1‐40 with all‐cause mortality partly mediated by GFR.

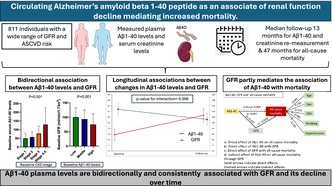

## 1 INTRODUCTION

Atherosclerotic cardiovascular disease (ASCVD) remains the leading cause of death worldwide^1^ and its prevention constitutes a continuous health challenge. In this context, effective reduction of ASCVD‐related mortality necessitates the identification of novel biological pathways which may refine ASCVD risk stratification and pinpoint new therapeutic targets. Within this framework, recent accumulating evidence indicates that amyloid‐beta (Αβ) metabolism, involved in Alzheimer's disease (AD) and amyloid cerebral angiopathy,^2^ plays a pivotal role in vascular aging and development of cardiovascular disease (CVD) including ASCVD and heart failure.^3^

Aβ peptides are generated by proteolytic cleavage of the amyloid precursor protein (APP) by beta‐ and gamma‐secretases^4^, ^5^ and exert potent proinflammatory and pro‐atherosclerotic properties.^3^, ^6^, ^7^ Aβ1‐40 is predominantly found in human atherosclerotic lesions^7^ where it activates proinflammatory sequelae in endothelial cells and macrophages involving cytokine secretion and oxidative stress leading to vascular disease.^6^, ^8^, ^9^ Notably, among other factors, renal dysfunction may increase circulating levels and subsequent tissue deposition of Aβ1‐40 by decreasing Aβ1‐40 clearance.^10^ Under normal conditions an equilibrium exists between Aβ1‐40 production and removal. Destabilization of this equilibrium may lead to accumulation of Aβ1‐40 in blood, vascular wall and heart tissues, encompassing enhanced risk for ASCVD.^3^ Αβ1‐40 levels have been previously associated with renal dysfunction with a concentration‐dependent manner with increasing plasma Αβ1‐40 across chronic kidney disease (CKD) stages.^11^, ^12^

It has been previously demonstrated that circulating Αβ1‐40 levels are associated with increased mortality in elderly patients, in patients with stable coronary artery disease (CAD) and in those with non‐ST‐segment elevation acute coronary syndrome (NSTE‐ACS), supporting a role of this peptide as a prognostic biomarker of CVD.^13^, ^14^, ^15^ However, Aβ1‐40 has been also associated with all‐cause death beyond ASCVD mortality.^16^ Previous evidence indicates accumulation of Aβ peptides in kidneys, as Aβ aggregates in renal tubular epithelial cells.^17^ However, whether Αβ1‐40 levels may longitudinally deteriorate renal function has not been investigated. The link between early renal dysfunction and high risk for all‐cause mortality is well established.^18^ Therefore, derangement in renal homeostasis associated with Αβ1‐40 levels may act synergistically towards other adverse outcomes mediated by Aβ1‐40 and may contribute to its association with all‐cause mortality. Given the intercorrelations among baseline Αβ1‐40 levels and renal function, we aimed to examine potential dynamic cross‐sectional and longitudinal associations of Aβ1‐40 levels with renal function in a population with a wide range of glomerular filtration rate (GFR). Furthermore, we aimed to explore the hypothesis that GFR may partly mediate the association of Αβ1‐40 with all‐cause mortality.

## 2 METHODS

### 2.1 Study population

#### 2.1.1 Athens Angiometabolic Registry Study

This is a registry consisting of two substudies: one cross‐sectional including all consecutively recruited patients (Substudy I), and one longitudinal part including consecutive patients who were followed for future events (Substudy II). This ongoing registry aims to stratify ASCVD risk in participants undergoing evaluation in primary or secondary ASCVD prevention settings. Recruitment is conducted at the Unit of Dyslipidemias and Atherosclerosis of the Department of Clinical Therapeutics, National and Kapodistrian University of Athens, as previously described.^19^ Our study design included participants from both substudies, as follows:

*Substudy I*. We performed a retrospectively designed post hoc analysis on participants consecutively recruited between November 2015 and September 2019. Participants were included if they had available Αβ1‐40 measurements and creatinine levels for GFR calculation. These criteria resulted in a total population of *n* = 811 patients, of whom one patient was on kidney dialysis.

*Substudy II*. Regarding the longitudinal part, from the 811 patients in Substudy I, participants who consented to be followed for future events through telephone contact and through inspection of their medical records were included (*n* = 654). Among them, *n* = 50 were lost to follow‐up and *n* = 604 were included in the longitudinal analysis for mortality. An additional analysis was conducted in a subgroup of these patients who could attend successive site visits for re‐evaluation of Aβ1‐40 and creatinine levels (*n* = 189) (Figure S1).

All participants provided written consent for participation in the registry. The current study was conducted according to the principles of the Declaration of Helsinki and the Local Ethics Committee of Alexandra General Hospital approved the study's protocol (13/26.11.2015).

Enrollment in the study required informed written consent, and baseline data were collected at the time of enrollment. All participants underwent comprehensive medical documentation, including history, clinical and laboratory exams to assess cardiovascular (CV) risk factors such as age, sex, body mass index (BMI), smoking, diabetes, hypertension, hyperlipidemia and renal function. Although the registry is ongoing, we specified an end‐of‐study date for Substudy I, set at the conclusion of February 2022. Eligible participants were adults referred for risk stratification for ASCVD and preventive therapy to our clinic for vascular studies or admitted for acute coronary syndrome (ACS). Exclusion criteria included individuals with life expectancy under 1 year (excluding ASCVD causes), severe valvular heart disease, acute or recent myocarditis (within 6 months), recent ACS (within 1 month), end‐stage renal disease, active malignancy or autoimmune/infectious diseases. Traditional risk factors (TRFs) were defined as previously described.^19^

### 2.2 Laboratory variables and Aβ1‐40 measurements

Fasting blood samples were acquired with venipuncture for standard biochemical lipid profile as well as creatinine levels. Abbreviated four‐variable Modification of Diet in Renal Disease (MDRD) formula was used to estimate GFR using the equation: 186 × [serum creatinine (mg/dl)] − 1.154 × (age) − .203 × (.742 if female).^20^ Plasma and serum samples for measurement of Αβ1‐40 levels were stored at −80°C until procession, whereas samples for biochemical profile measurements including creatinine levels were processed immediately after collection for analysis. Concentrations of Αβ1‐40 in ethylenediaminetetraacetic acid (EDTA)‐plasma samples were measured both in the baseline and follow‐up visit using a reliable enzyme‐linked immunosorbent assay (ELISA) kit manufactured by Biosource/Invitrogen in California, USA. The ELISA kit has been well‐characterized and previously described in the literature.^15^ The intra‐ and inter‐assay coefficient of variance of the ELISA measurements were reported to be less than 8%, and the minimum detectable concentration of human Aβ1‐40 was <6 pg/mL (Biosource/Invitrogen, California, USA). All measurements were performed by experienced personnel who were blinded to patients' characteristics. A uniform algorithm was followed for blood collection and plasma preparation for all patients in previously unthawed samples. To minimize systematic errors between the two time points, baseline and follow‐up samples from each participant were analysed simultaneously in pairs in the same ELISA plate.

### 2.3 Follow‐up and outcomes

All‐cause mortality was defined as the study main clinical endpoint. In patients who consented to be followed for future events, follow‐up evaluations were conducted annually either by site visit or by telephone contact if a site visit was not possible, as previously described.^19^ End‐of‐study date for the current study for follow‐up assessments (Substudy II) was set at the conclusion of September 2023. Median follow‐up time was 47 months whereas the maximum follow‐up was 76 months. Medical records were reviewed and evaluated by an independent clinician, blinded to both the CV risk profile and the CKD stage of participants, to adjudicate fatal events. All‐cause mortality was defined as the primary outcome of the study given that is it less subject to misinterpretation and subsequent detection bias than other endpoints.^21^

### 2.4 Statistical analysis

Continuous variables are shown as mean values ± standard deviation or median (interquartile range) for non‐normally distributed variables while categorical variables are presented as absolute values (count) and percentages. Normality of continuous variables was graphically assessed by histograms and P–P plots. The Independent *t*‐test and Mann–Whitney test were used to compare patients' characteristics in the lower versus highest tertile of Aβ1‐40 for continuous variables; the chi‐squared test was employed for categorical variables. To compare Αβ1‐40 or GFR across more than two groups, one‐way analysis of variance (ANOVA) was employed.^22^ Post‐ANOVA pairwise comparisons were conducted using Fisher's Least Significant Difference (LSD) test.^23^ Next, we employed regression analysis (i.e. linear regression and logistic regression) to evaluate the bidirectional independent association between Aβ1‐40 levels and GFR at baseline after adjusting for TRFs including age, sex, hypertension, hyperlipidemia, diabetes and smoking. Multivariable model was built based on biological plausibility. Estimated odds ratios (OR) and coefficients with the respective 95% confidence intervals (CI) were documented. Respectively, we implemented multivariable logistic and linear regression analysis to examine the association between changes in GFR between baseline and follow‐up and Aβ1‐40 tertiles—and vice versa‐ after adjustment for the core model. We also used linear and generalized linear mixed models with random intercept and unstructured variance–covariance matrix to examine simultaneous changes in Aβ1‐40 and GFR. Mixed models were adjusted for the same core model of TRFs.

Finally, we used structural equation modelling (SEM) to assess both direct and indirect effects (i.e. mediation analysis) of Aβ1‐40 and GFR on all‐cause mortality after controlling for confounders (TRFs). Indirect effects were quantified using the robust estimator of the variance–covariance (Huber/White/sandwich estimator). Path diagrams were used to visualize underlying SEM models with arrows indicating non‐causal relationships while single‐headed arrows denote unidirectional regression relationships. Only measured variables (depicted by squares in path diagrams) were used in the current one‐step SEM modelling. Selection of confounders in SEM was based on biological plausibility and previous literature. The comparative fit index and the root mean square error of approximation (RMSEA) were calculated to assess model fit for SEM analysis. To assess the proportion of the total effect attributable to the indirect pathway, we calculated the indirect‐to‐total effect ratio (ITR).^24^ This metric quantifies the relative contribution of the mediated effect to the overall effect of the exposure on the outcome. The ITR was defined as the ratio of the indirect effect to the total effect, expressed as ‘ITR, indirect effect/total effect.’

In terms of a priori power considerations, our study with 811 participants was adequately powered at .85 level to detect a 1.8‐fold increase in the odds of worsening renal function (CKD stage 3 or worse CKD stage at follow‐up compared with baseline) for subjects with high compared to those with lower baseline levels of Aβ1‐40 (highest vs. lower tertiles), assuming a reference incidence rate of 20%. In addition, we retained a ratio of 10 events to each covariate used as a rule of thumb in all multivariable logistic/Cox regression models.^25^ Missing values for key variables were <10% (Table S1) and no imputations were performed.^26^

Statistical analysis was conducted with SPSS version 29 (IBM SPSS Statistics, Inc., Chicago, IL) and STATA 18 (StataCorp LLC, Texas USA). We deemed statistical significance at *p* < .05. All tests were two‐tailed.

## 3 RESULTS

### 3.1 Demographic characteristics of the population according to Aβ1‐40 levels by tertiles

**FIGURE 1:**
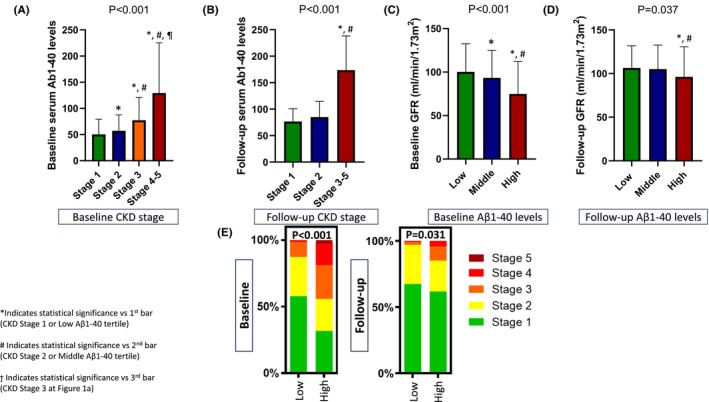
Consistent associations of Aβ1‐40 with GFR and CKD stage over time. Association of CKD stage with serum Aβ1‐40 levels (A) at baseline and (B) at follow‐up and association of Αβ1‐40 levels by tertiles (low, middle and high) with GFR (C) at baseline and (D) at follow‐up. (E) Transition by CKD stages according to Αβ1‐40 levels. Αβ1‐40, amyloid‐beta 1–40; CKD, chronic kidney disease; GFR, glomerular filtration rate. *p* value is derived by independent samples Student's *t*‐test for continuous variables and the chi‐squared test for categorical ones. Effect size corresponds to highest versus lower tertiles of Aβ1‐40 serum levels. Fisher's least significant difference (LSD) was implemented for pairwise comparisons.

Descriptive characteristics of the study population according to baseline serum Aβ1‐40 levels by tertiles are shown in Table 1. Patients with Aβ1‐40 levels at the highest tertile showed a significantly higher prevalence of CAD (24.7% vs. 17.0% vs. 8.9% for highest, middle and lowest tertile respectively, *p* < .001) and TRFs such as hypertension and diabetes (*p* < .05 for both) compared with their counterparts at lower tertiles (Table 1). Similarly, patients with increased Aβ1‐40 levels were older and exhibited significantly higher levels of systolic blood pressure (SBP) and aortic SBP (*p* < .001 for all, Table 1). Concerning the relationship between Aβ1‐40 levels and renal function, patients with Aβ1‐40 at the highest tertile had significantly worse renal function reflected as increased levels of creatinine (1.36 vs. .92 vs. .88 mg/dL for highest, middle and lowest tertile, respectively, *p* < .001), decreased GFR (74.8 vs. 93.3 vs. 100.2 mL/min/1.73m^2^ for highest, middle and lowest tertile, respectively, *p* < .001) and increased prevalence of stage 3–5 CKD (35.1% vs. 13.0% vs. 10.0% for highest, middle and lowest tertile respectively, *p* < .001) (Table 1). Interestingly, at a different time point (i.e. at 1‐year follow‐up), consistent associations of Aβ1‐40 with GFR over time were observed. Patients with Aβ1‐40 at the highest tertile had significantly increased creatinine levels, decreased GFR and increased prevalence of stage 3–5 CKD (*p* < .05 for all) (Figure 1). Descriptive characteristics of the population with follow‐up information between 1st and 2nd visiting time interval are shown in Table S2.

**TABLE 1:** Descriptive characteristics of the cohort population by tertiles of baseline serum Aβ1‐40 levels.

Variable	All	Aβ1‐40	Aβ1‐40	Aβ1‐40	*p*‐Value
		1st tertile	2nd tertile	3rd tertile	
	(*n* = 811)	(*n* = 270)	(*n* = 270)	(*n* = 271)	
Cardiometabolic risk factors					
Age (years)	**61.1 (12.0)**	**58.1 (11.6)**	**61.8 (11.9)**	**63.5 (11.8)**	**<.001**
Sex (male) (*n*, %)	**515 (63.5)**	**156 (57.8)**	**171 (63.3)**	**188 (69.4)**	.**035**
BMI (kg/m^2^)	28.0 (4.8)	28.2 (5.5)	27.7 (4.0)	28.1 (4.8)	.554
Smoking (*n*, %)	296 (36.5)	107 (39.6)	101 (37.4)	88 (32.5)	.240
Hypertension (*n*, %)	**438 (54.0)**	**128 (47.4)**	**142 (52.6)**	**168 (62.0)**	.**002**
Hyperlipidemia (*n*, %)	477 (58.8)	144 (53.3)	164 (60.7)	169 (62.4)	.066
Diabetes mellitus (*n*, %)	**186 (22.9)**	**51 (18.9)**	**54 (20.0)**	**81 (29.9)**	.**003**
Presence of CAD (*n*, %)	**137 (16.9)**	**24 (8.9)**	**46 (17.0)**	**67 (24.7)**	**<.001**
Statins (*n*, %)	**353 (43.5)**	**99 (36.7)**	**122 (45.2)**	**132 (48.7)**	.**014**
Antihypertensive treatment (*n*, %)	**457 (56.4)**	**123 (45.6)**	**153 (56.7)**	**181 (66.8)**	**<.001**
Antiplatelet treatment (*n*, %)	**162 (20.0)**	**35 (13.0)**	**55 (20.4)**	**72 (26.6)**	**<.001**
SBP (mmHg)	**131.9 (21.0)**	**127.8 (19.6)**	**131.0 (20.9)**	**136.6 (21.5)**	**<.001**
DBP (mmHg)	73.9 (11.3)	73.4 (11.7)	73.5 (10.8)	74.7 (11.4)	.392
Aortic SBP (mmHg)	**122.5 (20.8)**	**118.8 (19.5)**	**120.9 (19.5)**	**127.6 (22.1)**	**<.001**
Aortic DBP (mmHg)	74.1 (11.8)	74.4 (12.3)	73.4 (10.8)	74.5 (12.2)	.624
Fasting glucose (mg/dL)	109.3 (42.7)	104.4 (37.9)	110.6 (37.1)	112.8 (51.3)	.079
Total cholesterol (mg/dL)	194.6 (45.9)	198.3 (43.4)	196.7 (42.7)	188.9 (50.7)	.056
HDL‐C (mg/dL)	**51.2 (17.4)**	**52.5 (16.7)**	**54.3 (19.6)**	**46.7 (14.8)**	**<.001**
LDL‐C (mg/dL)	124.2 (46.8)	127.3 (54.5)	124.6 (39.4)	120.6 (44.2)	.314
Triglycerides (mg/dL)	127.8 (72.1)	123.1 (75.7)	124.8 (59.1)	135.6 (79.2)	.126
Aβ1‐40 level (pg/mL)	**60.1 (42.9)**	**25.3 (11.7)**	**53.2 (6.6)**	**101.8 (42.2)**	**<.001**
Creatinine (mg/dL)	**1.05 (.77)**	.**88 (.60)**	.**92 (.41)**	**1.36 (1.05)**	**<.001**
GFR (mL/min/1.73m^2^)	**89.3 (35.5)**	**100.2 (32.3)**	**93.3 (31.7)**	**74.8 (37.4)**	**<.001**
GFR <60 mL/min/1.73m^2^ (*n*, %)	**157 (19.4)**	**27 (10.0)**	**35 (13.0)**	**95 (35.1)**	**<.001**

*Note*: Continuous variables are presented as mean (SD) and nominal as count (absolute percentages). *p*‐value is derived by independent samples Student's *t*‐test for continuous variables and the chi‐squared test for categorical ones. Boldface values indicate statistical significance, which was set at the level of *p*‐value <.05.

Abbreviations: Aβ1‐40, amyloid‐beta 1–40; BMI, body mass index; CAD, coronary artery disease; DBP, diastolic blood pressure; GFR, glomerular filtration rate; HDL‐C, high‐density lipoprotein cholesterol; LDL‐C, low‐density lipoprotein cholesterol; SBP, systolic blood pressure.

### 3.2 Intercorrelations between Aβ1‐40 levels and renal function at baseline and follow‐up

We found bidirectional associations of Aβ1‐40 levels with GFR as demonstrated in Table 2. After adjustment for TRFs including age, sex, smoking, hypertension, diabetes and hyperlipidemia, continuous Aβ1‐40 levels were inversely associated with GFR (*β*‐coefficient = −.217, *p* < .001). Furthermore, after adjustment for the core model including the same TRFs, Aβ1‐40 at the highest tertile was associated with more than 2.2‐fold higher odds for stage ≥2 (OR = 2.29, *p* < .001) and more than 3.6‐fold higher risk for stage 3–5 CKD (OR = 3.67, *p* < .001) (Table 2). On the contrary, baseline GFR also demonstrated a significant inverse association with baseline Aβ1‐40 levels (adjusted *β*‐coefficient = −.449, *p* < .001) (Table 2).

**TABLE 2:** Bidirectional associations of baseline serum Aβ1‐40 levels with baseline renal function.

Entire study population (*N* = 811)						
Regression of Aβ1‐40 levels on GFR (dependent: GFR)						
	Association between continuous Aβ1‐40 levels and GFR		Association between high Aβ1‐40* and GFR≤90 mL/min/1.73m^2^		Association between high Aβ1‐40* and GFR≤60 mL/min/1.73m^2^	
Exposure variables	*β*‐coefficient (95% CI)	*p*‐Value	OR (95% CI)	*p*‐Value	OR (95% CI)	*p*‐Value
Univariable (Aβ1‐40)	**−.302 (−.357, −.248)**	**<.001**	**2.73 (2.00, 3.73)**	**<.001**	**4.18 (2.90, 6.04)**	**<.001**
+ Age	**−.247 (−.297, −.198)**	**<.001**	**2.53 (1.78, 3.60)**	**<.001**	**3.90 (2.62, 5.80)**	**<.001**
+ Sex (male)	**−.291 (−.344, −.238)**	**<.001**	**2.65 (1.93, 3.64)**	**<.001**	**4.03 (2.78, 5.84)**	**<.001**
+ Diabetes	**−.271 (−.325, −.217)**	**<.001**	**2.53 (1.83, 3.54)**	**<.001**	**3.88 (2.64, 5.72)**	**<.001**
+ Smoking	**−.293 (−.347, −.239)**	**<.001**	**2.63 (1.91, 3.61)**	**<.001**	**4.06 (2.78, 5.95)**	**<.001**
+ Hyperlipidemia	**−.285 (−.338, −.232)**	**<.001**	**2.67 (1.94, 3.67)**	**<.001**	**4.03 (2.75, 5.88)**	**<.001**
+ Hypertension	**−.243 (−.293, −.193)**	**<.001**	**2.53 (1.80, 3.56)**	**<.001**	**3.90 (2.60, 5.84)**	**<.001**
+ hs‐CRP	**−.268 (−.326, −.210)**	**<.001**	**2.61 (1.86, 3.65)**	**<.001**	**3.35 (2.22, 5.06)**	**<.001**
Multivariable model 1^a^	**−.217 (−.264, −.169)**	**<.001**	**2.29 (1.58, 3.31)**	**<.001**	**3.67 (2.37, 5.70)**	**<.001**
Multivariable model 2^b^	**−.183 (−.234, −.132)**	**<.001**	**2.12 (1.42, 3.16)**	**<.001**	**2.54 (1.55, 4.17)**	**<.001**

Regression of GFR on Aβ1‐40 levels (dependent: Aβ1‐40)						
	Association between GFR and Aβ1‐40 levels		Association between GFR≤90 mL/min/1.73m^2^ and high Aβ1‐40* levels		Association between GFR≤60 mL/min/1.73m^2^ and high Aβ1‐40* levels	
Exposure variables	*β*‐coefficient (95% CI)	*p*‐Value	OR (95% CI)	*p*‐Value	OR (95% CI)	*p*‐Value
Univariable (GFR)	**−.450 (−.531, −.370)**	**<.001**	**2.73 (2.00, 3.73)**	**<.001**	**4.18 (2.90, 6.04)**	**<.001**
+ Age	**−.454 (−.531, −.370)**	**<.001**	**2.53 (1.78, 3.59)**	**<.001**	**3.86 (2.60, 5.73)**	**<.001**
+ Sex (male)	**−.458 (−.541, −.375)**	**<.001**	**2.65 (1.93, 3.64)**	**<.001**	**4.03 (2.78, 5.84)**	**<.001**
+ Diabetes	**−.425 (−.510, −.340)**	**<.001**	**2.53 (1.83, 3.48)**	**<.001**	**3.88 (2.64, 5.72)**	**<.001**
+ Smoking	**−.448 (−.531, −.365)**	**<.001**	**2.63 (1.91, 3.61)**	**<.001**	**4.06 (2.78, 5.95)**	**<.001**
+ Hyperlipidemia	**−.451 (−.536, −.367)**	**<.001**	**2.67 (1.94, 3.67)**	**<.001**	**4.03 (2.75, 5.88)**	**<.001**
+ Hypertension	**−.450 (−.542, −.358)**	**<.001**	**2.53 (1.80, 3.56)**	**<.001**	**3.90 (2.60, 5.84)**	**<.001**
+ hs‐CRP	**−.412 (−.501, −.323)**	**<.001**	**2.51 (1.80, 3.52)**	**<.001**	**3.35 (2.22, 5.06)**	**<.001**
Multivariable model 1^a^	**−.449 (−.548, −.350)**	**<.001**	**2.31 (1.60, 3.34)**	**<.001**	**3.52 (2.30, 5.40)**	**<.001**
Multivariable model 2^b^	**−.388 (−.497, −.279)**	**<.001**	**2.05 (1.37, 3.06)**	**<.001**	**2.51 (1.55, 4.06)**	**<.001**

*Note*: Boldface values indicate statistical significance, which was set at the level of *p*‐value <.05.

Abbreviations: Aβ1‐40, amyloid‐beta 1–40; CI, confidence intervals; GFR, glomerular filtration rate; hs‐CRP, high sensitivity C‐reactive protein; OR, odds ratio.

* Effect size corresponds to highest versus lower tertiles of Aβ1‐40 serum levels.

^a^ Adjusted for age, sex, smoking, hypertension, hyperlipidemia and diabetes mellitus.

^b^ Adjusted for age, sex, smoking, hypertension, hyperlipidemia, diabetes mellitus and hs‐CRP.

When assessing the same association at a different time point, at 1‐year follow‐up, similar associations were observed (Figure 1). In detail, continuous Aβ1‐40 levels were inversely associated with GFR (adjusted *β*‐coefficient = −.185, *p* = .003) and vice versa (adjusted *β*‐coefficient = −.317, *p* < .001).

### 3.3 Longitudinal bidirectional association of Aβ1‐40 levels with renal function at follow‐up

**FIGURE 2:**
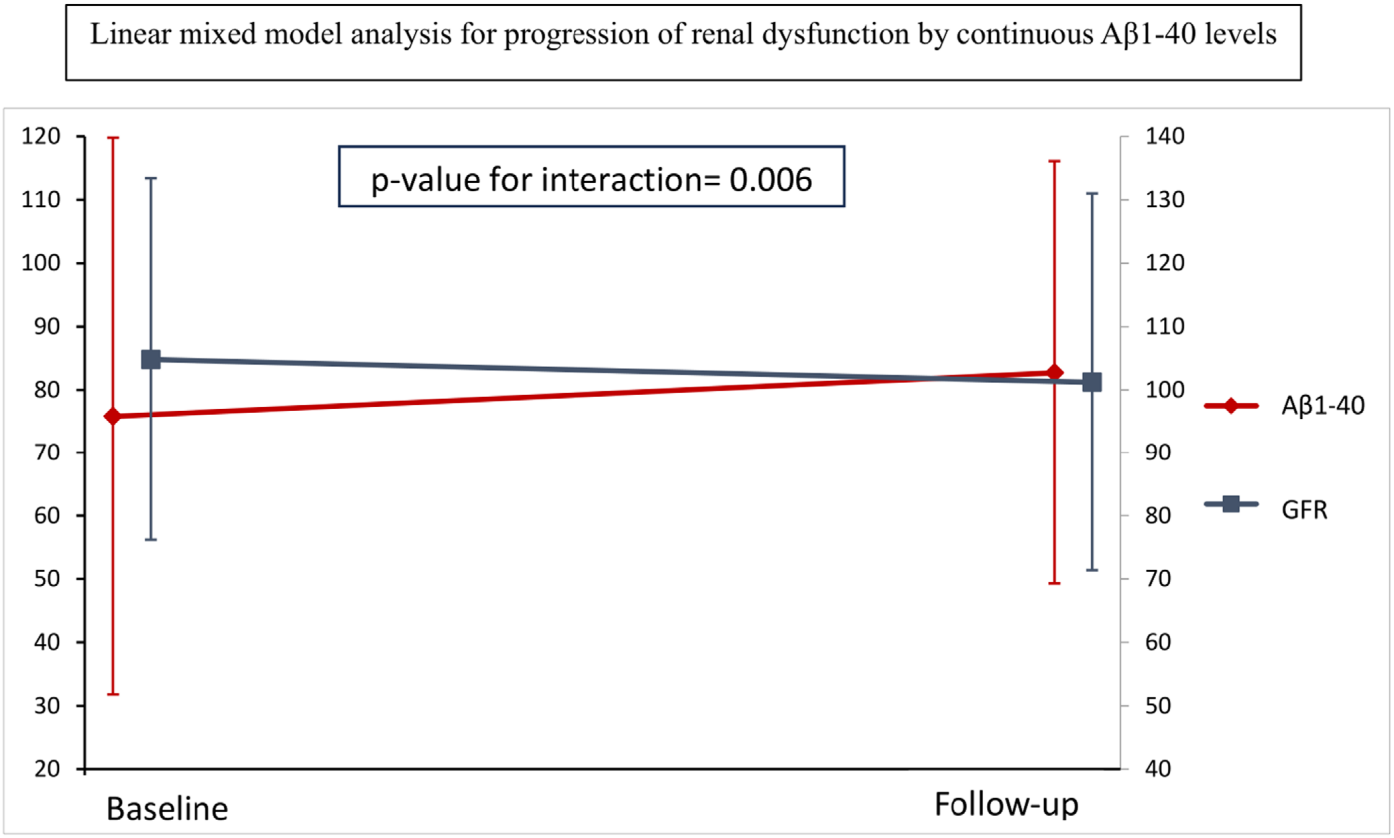
Longitudinal associations between changes in Αβ1‐40 levels and GFR. Right axis corresponds to GFR values whereas left axis corresponds to Αβ1‐40 levels. Aβ1‐40, amyloid‐beta 1–40; GFR, glomerular filtration rate.

Similarly, we found bidirectional longitudinal associations of Aβ1‐40 levels with GFR as demonstrated in Table 3. After a median follow‐up period of 13 months, baseline Aβ1‐40 levels were inversely associated with GFR values at follow‐up after adjustment for TRFs (*β*‐coefficient = −.151, *p* < .001) (Table 3). Moreover, Aβ1‐40 levels at the highest tertile at baseline were associated with more than 2.2‐fold increased risk for CKD stage 3 or worse CKD stage at follow‐up compared with baseline (adjusted OR = 2.26, *p* = .033). GFR at baseline was also inversely associated with Aβ1‐40 levels at follow‐up (*β*‐coefficient = −.423, *p* < .001) (Table 3). By linear mixed model analysis, changes in Aβ1‐40 concentration were inversely associated with changes in GFR across the follow‐up period [OR = .77 per 1 standard deviation (SD) increase in Aβ1‐40, *p* = .006] whereas this increase was also associated with almost 2.5‐fold increased odds for a worse CKD stage at follow‐up (OR = 2.49, *p* < .001 per 1 SD increase in Aβ1‐40 concentration) (Figure 2).

**TABLE 3:** Longitudinal bidirectional association of Aβ1‐40 levels with renal function after a median follow‐up time of 13 months.

Entire study population with follow‐up (*N* = 189)				
Exposure variables	*β*‐coefficient (95% CI)	*p*‐Value	OR (95% CI)	*p*‐Value
Regression of Aβ1‐40 levels on GFR (dependent: GFR)				
	Association between continuous baseline Aβ1‐40 levels and GFR at follow‐up		Association between Aβ1‐40* at baseline and GFR ≤60 mL/min/1.73m^2^ or worsening CKD stage at follow‐up	
Univariable (Aβ1‐40)	**−.167 (−.248, −.086)**	**<.001**	**2.17 (1.04, 4.53)**	.**038**
+ Age	**−.157 (−.235, −.078)**	**<.001**	**2.15 (1.03, 4.48)**	.**042**
+ Sex (male)	**−.164 (−.245, −.082)**	**<.001**	**2.17 (1.04, 4.53)**	.**038**
+ Diabetes	**−.167 (−.249, −.086)**	**<.001**	**2.18 (1.05, 4.56)**	.**038**
+ Smoking	**−.170 (−.250, −.089)**	**<.001**	**2.20 (1.06, 4.60)**	.**035**
+ Hyperlipidemia	**−.167 (−.248, −.086)**	**<.001**	**2.21 (1.06, 4.63)**	.**035**
+ Hypertension	**−.162 (−.242, −.082)**	**<.001**	**2.25 (1.07, 4.71)**	.**032**
+ hs‐CRP	**−.178 (−.261, −.095)**	**<.001**	**2.18 (1.03, 4.64)**	.**043**
Multivariable model 1^a^	**−.151 (−.229, −.073)**	**<.001**	**2.26 (1.07, 4.79)**	.**033**
Multivariable model 2^b^	**−.168 (−.248, −.088)**	**<.001**	**2.15 (1.00, 4.62)**	.**049**

Regression of GFR on Aβ1‐40 levels (dependent: Aβ1‐40)				
	Association between GFR at baseline and Aβ1‐40 levels at follow‐up		Association between GFR ≤90 mL/min/1.73m^2^ at baseline and high Aβ1‐40* levels at follow‐up	
Univariable (GFR)	**−.427 (−.605, −.249)**	**<.001**	**2.54 (1.25, 5.15)**	.**010**
+ Age	**−.359 (−.548, −.170)**	**<.001**	**2.15 (1.03, 4.50)**	.**042**
+ Sex (male)	**−.428 (−.606, −.250)**	**<.001**	**2.49 (1.23, 5.06)**	.**011**
+ Diabetes	**−.437 (−.615, −.259)**	**<.001**	**2.67 (1.30, 5.48)**	.**007**
+ Smoking	**−.460 (−.644, −.275)**	**<.001**	**2.93 (1.40, 6.14)**	.**004**
+ Hyperlipidemia	**−.426 (−.605, −.248)**	**<.001**	**2.62 (1.29, 5.32)**	.**008**
+ Hypertension	**−.412 (−.588, −.235)**	**<.001**	**2.32 (1.12, 4.76)**	.**023**
+ hs‐CRP	**−.440 (−.627–.253)**	**<.001**	**2.82 (1.34, 5.92)**	.**006**
Multivariable model 1^a^	**−.423 (−.616, −.230)**	**<.001**	**2.65 (1.21, 5.81)**	.**015**
Multivariable model 2^b^	**−.441 (−.643, −.240)**	**<.001**	**2.27 (1.13, 4.57)**	.**022**

*Note*: Boldface values indicate statistical significance, which was set at the level of *p*‐value <.05.

Abbreviations: Aβ1‐40, amyloid‐beta 1–40; CI, confidence intervals; GFR, glomerular filtration rate; hs‐CRP, high sensitivity C‐reactive protein; OR, odds ratio.

* Effect size corresponds to highest versus lower tertiles of Aβ1‐40 serum levels.

^a^ Adjusted for age, sex, smoking, hypertension, hyperlipidemia and diabetes mellitus.

^b^ Adjusted for age, sex, smoking, hypertension, hyperlipidemia, diabetes mellitus and hs‐CRP.

### 3.4 Mediation analysis

**FIGURE 3:**
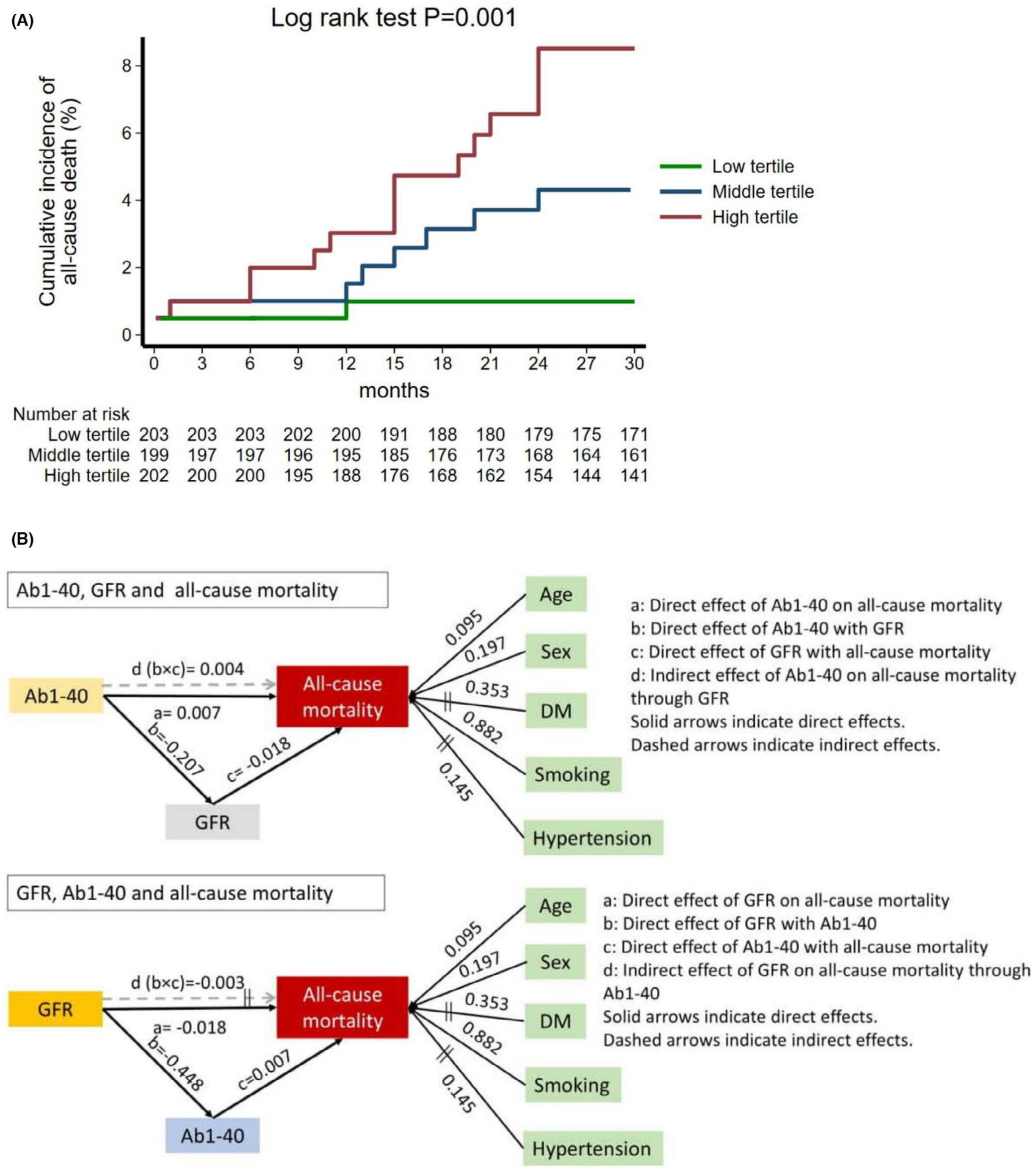
(A) Nelson Aalen curves for the cumulative incidence of the endpoint of all‐cause death according to tertiles of Αβ1‐40 (low, middle and high) across the follow‐up period. Seven out of 223 patients (3.1%) with Αβ1‐40 level at the low tertile versus 12 out of 199 patients (6.0%) with Αβ1‐40 level at the middle tertile versus 25 out of 203 patients (12.3%) with Αβ1‐40 level at the high tertile suffered a fatal event. Nelson‐Aalen plots were derived for 604 patients with available both Αβ1‐40 levels at baseline and follow‐up data. The Nelson‐Aalen plots depict the cumulative hazard rate function from survival data across a time period, separately for each group of interest (i.e. high vs. middle vs. low tertile of Αβ1‐40). The risk table beneath these plots shows the number of patients at risk of experiencing the event for each time. (B) Pathway modelling of longitudinal inter‐relationships among Aβ1‐40 levels, GFR and all‐cause mortality by SEM analysis after controlling for pre‐specified confounders. Aβ1‐40 exerted direct (a) and indirect effect (d) on all‐cause mortality mediated through GFR. Multi‐level (2‐level) generalized structural equation models were used to derive statistical inference. Standardized regression coefficients (parameter weights) are shown adjacent to each path. Grey dashed lines indicate the indirect effect of Aβ1‐40 on all‐cause mortality through GFR. Non‐significant paths are marked with two vertical lines (i.e. ||) in their middle. Αβ1‐40, amyloid‐beta 1–40; DM, diabetes mellitus; GFR, glomerular filtration rate.

Over a median period of 47 months, 44 deaths were recorded. Aβ1‐40 was associated with all‐cause mortality after adjustment for the core model including TRFs [hazard ratio (HR) = 1.20 per 1 SD increase in Aβ1‐40, *p* = .016] (Figure 3). Mediation analysis showed both a direct (OR = 1.37, *p* = .045 per 1 SD increase in Αβ1‐40) and an indirect effect of Aβ1‐40 on its association with all‐cause mortality, mediated through GFR (OR = 1.20, *p* = .020 per 1 SD increase in Αβ1‐40) (Figure 3). In our analysis, the estimated ITR was .334 (95% CI .021–.647, *p* = .037), indicating that 33.4% of the total effect of Αβ1‐40 on all‐cause mortality operates through the mediated pathway involving GFR. In contrast, we did not find an indirect effect of GFR on all‐cause mortality mediated through Aβ1‐40 (*p* > .05) (Figure 3).

## 4 DISCUSSION

In the present study, we comprehensively investigated the nature and direction of the association between Αβ1‐40 levels and renal function in a population with a wide range of ASCVD risk. We identified a robust and independent bidirectional association between Αβ1‐40 levels and GFR. This association was longitudinally consistent over two long‐term successive visits. Longitudinal changes were intercorrelated while increased Aβ1‐40 levels at baseline predicted progression of CKD stage at follow‐up. Importantly, exploring the clinical relevance of these observations, we found that the association of high Aβ1‐40 levels with all‐cause mortality was partly mediated by its association with GFR. To assess the proportion of the total effect attributable to the indirect pathway of GFR in the association of Αβ1‐40 with all‐cause mortality, we calculated the ITR and found that 33.4% of the total effect of Αβ1‐40 on all‐cause mortality operates through the mediated pathway involving GFR, suggesting a substantial role for GFR in explaining the observed relationship.

In our study, Αβ1‐40 levels were associated with lower GFR values both at baseline and at follow‐up in a population with diverse ASCVD risk. Previous evidence supports our findings indicating an association between Αβ1‐40 levels and renal dysfunction.^10^, ^11^ Indeed, Αβ1‐40 clearance seems to be largely dependent on renal function, which in turn has an impact on Aβ1‐40 levels.^3^ Conversely, Αβ1‐40 is found in renal tubular epithelial cells.^17^ APP and its cleavage enzyme beta‐secretase 1 (BACE1), which generate Aβ peptides, have been found in the epithelial renal tubular cells of APP23 mice where they were associated with worse renal pathology.^27^ Studies suggest that Αβ1‐40 can accumulate in vascular and parenchymal tissues, contributing to endothelial dysfunction, oxidative stress and fibrosis.^28^ Such mechanisms could also affect the kidneys, leading to disruptions in renal microvasculature and filtration processes.^28^, ^29^, ^30^, ^31^ However, further research is needed in humans to specifically investigate the role of Aβ1‐40 in renal pathology, including its potential accumulation and damage to renal tissues. Importantly, Aβ abnormal increased expression has been also observed in CKD patients, which undoubtedly contributes to pathological changes within the brain and accelerates cognitive decline.^32^ In brains of mice overexpressing APP, the expression level of BACE1 is increased, which in turn promotes Aβ deposition in the brain.^27^ Similarly, the expression of BACE1 in the brain and cerebrospinal fluid is also increased with the accumulation of Aβ plaques in the brain in AD patients.^33^ Interestingly, BACE1 is also involved in the pathological process of other diseases including CVD, given that the dysregulation of the BACE1/Beta‐Secretase‐1 Antisense RNA (BACE1‐AS)/Aβ axis may contribute to the pathophysiology of heart failure.^34^ In hypertensive rats, a strong association between CKD and cerebral Aβ pathology has been demonstrated whereas perivascular Aβ deposits were also detected in the direct vicinity of small vessel wall damage.^35^ Histological findings have confirmed the presence of Aβ1‐40 and ‐42 aggregates in the heart of AD patients.^36^ Collectively, these findings pinpoint the essential role of Αβ peptides in the diseases of the brain‐heart‐kidney axis. However, the clinical relevance of these findings has not been explored. Our study reports for the first time, that Αβ1‐40 is an independent determinant of GFR at two successive time points and of declining renal function over a median follow‐up period of 13 months, suggesting that, from a clinical perspective, not only renal dysfunction may lead to higher Αβ1‐40 levels, but a deteriorating effect of Αβ1‐40 on renal function may exist. This should be further investigated in preclinical studies.

Importantly, it has been previously shown that Aβ1‐40 exerts detrimental effects in multiple processes of ASCVD including endothelial cell activation, vascular smooth muscle cell contractility, monocyte adhesion, migration and transformation, platelet activation and aggregation.^3^ Within this framework, we have shown that higher circulating levels of Αβ1‐40 are associated with increased mortality in NSTE‐ACS and chronic coronary syndrome (CCS) patients adding reclassification value over established risk scores and risk factors.^14^, ^15^ In this context, to provide answers on potential mediators which could explain these associations we sought to investigate the association of Αβ1‐40 and GFR on all‐cause mortality. Notwithstanding, observational cross‐sectional studies cannot prove causative associations and are biased to reverse causality. For that purpose, to address this limitation we implemented mediation analysis and provided the first clinical hypothesis generating evidence of a pathway through which Αβ1‐40 portends increased risk for mortality. Our findings expand previous evidence and demonstrate that the deleterious effect of Αβ1‐40 on all‐cause mortality is partly mediated through renal dysfunction. Therefore, taking into consideration all the aforementioned, a vicious circle between renal function and Αβ1‐40 might exist which could act mutually and also additively towards the adverse prognosis of patients, given that both Αβ1‐40 and renal dysfunction have been associated with increased mortality.^14^, ^15^, ^18^

Our study raises clinical implications. Circulating Aβ1‐40 assessed with Aβ1‐42 is considered an early biomarker of high risk for dementia development.^37^ Similarly, accumulating preclinical and clinical evidence from our group, strongly support the clinical role of circulating Αβ1‐40 as a biomarker of adverse prognosis and risk stratification for CVD development and progression.^14^, ^15^ In the current study, we provide the first hypothesis generating data supporting a clinical role of circulating Aβ1‐40 as marker of renal function decline over time, which may partly mediate its association with increased mortality. Taken together, our findings support the hypothesis that Aβ1‐40 and possibly other components of amyloid metabolism may be clinically useful as a biomarker in the diseases of the brain‐heart‐kidney axis, playing the role of a common denominator in their pathophysiology. To that end, the clinical relevance of this hypothesis is further enhanced by emerging data showing that several established therapeutic interventions may exert off‐target effects on amyloid metabolism and Aβ1‐40 tissue and plasma levels. For example, statins and antihypertensive regimens affect APP processing and Αβ turnover, whereas haemodialysis directly reduces Αβ1‐40 levels.^3^

Several limitations should be acknowledged. First, the design of this study being a non‐interventional observational study hinders direct inference of causality. Further research is warranted to explore the validity and generalisability of these findings to other populations. Additional studies in community‐based cohorts are likely to be valuable in corroborating our findings. While the design of the study was predefined, the high attrition rate from the initial participants to those who could attend successive visits for re‐evaluation of Αβ1‐40 and creatinine levels should be acknowledged as a limitation. However, Αβ1‐40 levels in these patients remained inversely associated with GFR at a different time point (i.e. follow‐up visit), confirming the baseline findings.

In conclusion, the present study demonstrates that a pattern of continuously high or increasing Αβ1‐40 levels is independently and bidirectionally associated with GFR over two long‐term successive visits and may predict progression of CKD stage at follow‐up in a population with a wide range of ASCVD risk. Interestingly, it was also shown that longitudinal changes of Αβ1‐40 and GFR were intercorrelated. Moreover, the deleterious effect of Αβ1‐40 on all‐cause mortality was partly mediated through GFR. These findings suggest a mechanistic link between Aβ1‐40 and renal function and warrant further research to clarify the clinical value of monitoring its circulating levels as a novel biomarker of renal dysfunction.

## AUTHORS' CONTRIBUTIONS

GM, GG, KSte and KSta were involved in data curation. GM and GG were involved in formal analysis and methodology. GM was involved in writing—original draft. KSte and KSta were involved in funding acquisition and resources. KSta was involved in supervision. Each author contributed important intellectual content during manuscript drafting or revision and agrees to be personally accountable for the individual's own contributions and to ensure that questions pertaining to the accuracy or integrity of any portion of the work, even one in which the author was not directly involved, are appropriately investigated and resolved, including with documentation in the literature if appropriate.

## FUNDING INFORMATION

Dr. Georgiopoulos was supported by the Onassis foundation under the special Grant & support program for Scholars' Association Members (Grant No. R ZP 001/2019–2020). ST‐C is supported by the British Heart Foundation (PG/23/11093) and the Royal Society (RG\R1\241197). KSte has been supported by grants from the European Research Council (ERC) under the European Union's Horizon 2020 research and innovation programme (MODVASC, grant agreement No 759248), and is supported by the German Research Foundation DFG (CRC1366 C07, project number 394046768), the Health+Life Science Alliance Heidelberg Mannheim GmbH and the Helmholtz Institute for Translational AngioCardioScience (HI‐TAC).

## CONFLICT OF INTEREST STATEMENT

There are no additional relationships to disclose. There are no patents to disclose. There are no additional activities to disclose. All other authors have no conflicting interests to declare in regards to this manuscript.

## Supporting information


Appendix S1.

## Data Availability

Anonymized data can be requested after publication of the results of prespecified analyses from the corresponding authors to be shared subject to approval of institutional review boards.
