# Supplementary material for: Circulating amyloid beta 1‐40 peptide as an associate of renal function decline
Source: Eur J Clin Invest. 2025 Feb 24;55(5):e70006. doi: 10.1111/eci.70006 (PMC12011680; doi:10.1111/eci.70006)
Supplement: Supplementary file 1 — Appendix S1. [file ECI-55-e70006-s001.docx]

**Circulating amyloid beta 1-40 peptide as an associate of renal function decline mediating increased mortality.**

Georgios Mavraganis^1,*^, Georgios Georgiopoulos^1,2,*^, Georgios Zervas^1^, Evmorfia Aivalioti^1^, Dimitrios Delialis^1^, Ioannis Petropoulos^1^, Nikolaos Rachiotis^1^, Christina Konstantaki^1^, Chrysoula Moustou^1^, Maria-Aggeliki Dimopoulou^1^, Marco Sachse^3,4^, Simon Tual-Chalot^5^, Kateryna Sopova^4,6^, Erasmia Psimmenou^1^, Konstantinos Stellos^4-8,**^, Kimon Stamatelopoulos^1,5,**^

^1^Department of Clinical Therapeutics, Alexandra Hospital, National and Kapodistrian University of Athens Medical School, 80 Vas. Sofias Str, Athens 11528, Greece.

^2^Department of Physiology, School of Medicine, University of Patras, Patras, Greece.

^3^Department of Cardiovascular Surgery, University Heart and Vascular Centre, University Medical Centre Hamburg-Eppendorf, Hamburg, Germany

^4^Department of Cardiovascular Research, Medical Faculty Mannheim, Heidelberg University, Mannheim, Germany.

^5^Biosciences Institute, Vascular Biology and Medicine Theme, Faculty of Medical Sciences, Newcastle University, Newcastle upon Tyne, United Kingdom.

^6^Department of Cardiology, Angiology, Haemostaseology and Medical Intensive Care, University Medical Centre Mannheim, Heidelberg University, Mannheim, Germany.

^7^German Centre for Cardiovascular Research (DZHK), Partner Site Heidelberg/Mannheim, Mannheim, Germany.

^8^Helmholtz Institute for Translational AngioCardioScience (HI-TAC), Mannheim, Germany.

^*^ contributed equally
^**^ last-equal authors

**Address for Correspondence**

Kimon Stamatelopoulos, MD, PhD, Department of Clinical Therapeutics, Alexandra Hospital, Medical School, National and Kapodistrian University of Athens, PO Box 11528, 80 Vas. Sofias Str, Athens, Greece, Email: kstamatel@med.uoa.gr, Tel. +30-2132162487, Fax. +30-2103381487.

| **Supplementary Table 1. Percentage of missing data in the cohort** | | |
| --- | --- | --- |
| **Variable** | **Missing cases** | |
|  | **n** | **%** |
| **Age (years)** | 0 | 0 |
| **Sex (male) (n, %)** | 0 | 0 |
| BMI (kg/m^2^) | 79 | 9.7 |
| Smoking (n, %) | 21 | 2.6 |
| **Hypertension (n, %)** | 17 | 2.1 |
| Hyperlipidemia (n, %) | 19 | 2.3 |
| **Diabetes mellitus (n, %)** | 18 | 2.2 |
| **Presence of CAD (n, %)** | 0 | 0 |
| **Statins (n, %)** | 27 | 3.3 |
| **Antihypertensive treatment (n, %)** | 26 | 3.2 |
| **Antiplatelet treatment (n, %)** | 27 | 3.3 |
| **SBP (mmHg)** | 73 | 9.0 |
| DBP (mmHg) | 73 | 9.0 |
| Fasting glucose (mg/dl) | 78 | 9.6 |
| Total cholesterol (mg/dl) | 77 | 9.5 |
| **HDL-C (mg/dl)** | 80 | 9.9 |
| LDL-C (mg/dl) | 79 | 9.7 |
| Triglycerides (mg/dl) | 79 | 9.7 |
| **Aβ1-40 level (pg/mL)** | 0 | 0 |
| **Creatinine (mg/dl)** | 0 | 0 |
| **GFR (ml/min/1.73m^2^)** | 0 | 0 |
| Abbreviations: BMI, body mass index; CAD, coronary artery disease; SBP, systolic blood pressure; DBP, diastolic blood pressure; HDL-C, high-density lipoprotein cholesterol; LDL-C, low-density lipoprotein cholesterol; Aβ1-40, amyloid-beta 1-40; GFR, glomerular filtration rate. | | |

**Supplementary Table 2. Descriptive characteristics of the population with follow-up information between 1^st^ and 2^nd^ visiting time interval (n=189)**

| **Variable** | **Baseline** | **Follow-up** | **P-value** | |
| --- | --- | --- | --- | --- |
| **Cardiometabolic risk factors** | | | |  |
| Age (years) | 59.2 (11.7) | 60.4 (11.7) | 0.306 | |
| BMI (kg/m^2^) | 28.1 (4.8) | 28.3 (4.8) | 0.707 | |
| Waist-to-hip ratio | 0.97 (0.08) | 0.98 (0.10) | 0.444 | |
| Smoking (n, %) | 70 (37.0) | 62 (32.8) | 0.604 | |
| Hypertension (n, %) | 105 (55.6) | 109 (57.7) | 0.551 | |
| Hyperlipidemia (n, %) | 186 (98.4) | 187 (98.9) | 0.653 | |
| Diabetes mellitus (n, %) | 60 (31.7) | 61 (32.3) | 0.856 | |
| Presence of CAD (n, %) | 92 (48.7) | 92 (48.7) | 0.990 | |
| **Statins (n, %)** | **111 (58.7)** | **151 (79.9)** | **<0.001** | |
| Antihypertensive treatment (n, %) | 117 (61.9) | 126 (66.7) | 0.334 | |
| Antiplatelet treatment (n, %) | 92 (48.7) | 93 (49.2) | 0.878 | |
| B-blockers (n, %) | 80 (42.3) | 85 (45.0) | 0.604 | |
| RAAS inhibitors (n, %) | 85 (45.0) | 93 (49.2) | 0.410 | |
| SBP (mmHg) | 127.1 (18.1) | 126.3 (16.0) | 0.664 | |
| DBP (mmHg) | 72.5 (10.5) | 73.5 (10.4) | 0.374 | |
| Aortic SBP (mmHg) | 119.1 (18.7) | 118.9 (17.2) | 0.941 | |
| Aortic DBP (mmHg) | 72.5 (10.3) | 77.2 (54.4) | 0.251 | |
| Fasting glucose (mg/dl) | 105.8 (32.3) | 102.3 (24.8) | 0.236 | |
| **Total cholesterol (mg/dl)** | **175.2 (60.0)** | **150.0 (47.8)** | **<0.001** | |
| HDL-C (mg/dl) | 48.1 (15.6) | 48.7 (14.9) | 0.708 | |
| **LDL-C (mg/dl)** | **112.6 (54.8)** | **84.1 (44.4)** | **<0.001** | |
| Triglycerides (mg/dl) | 129.7 (76.4) | 121.1 (84.4) | 0.303 | |
| **Aβ1-40 level (pg/mL)** | **75.8 (28.0)** | **82.7 (33.4)** | **0.030** | |
| **Creatinine (mg/dl)** | **0.79 (0.53)** | **0.87 (0.79)** | **0.001** | |
| **GFR (ml/min/1.73m^2^)** | **105.9 (28.6)** | **101.3 (22.5)** | **<0.001** | |
| GFR<60 ml/min/1.73m^2^ (n, %) | 6 (3.3) | 13 (7.1) | 0.110 | |
| Note: Continuous variables are presented as mean (SD) and nominal as count (absolute percentages). Boldface values indicate statistical significance, which was set at the level of p-value <0.05.  Abbreviations: BMI, body mass index; CAD, coronary artery disease; RAAS, renin-angiotensin-aldosterone system; SBP, systolic blood pressure; DBP, diastolic blood pressure; HDL-C, high-density lipoprotein cholesterol; LDL-C, low-density lipoprotein cholesterol; GFR, glomerular filtration rate. | | | |  |


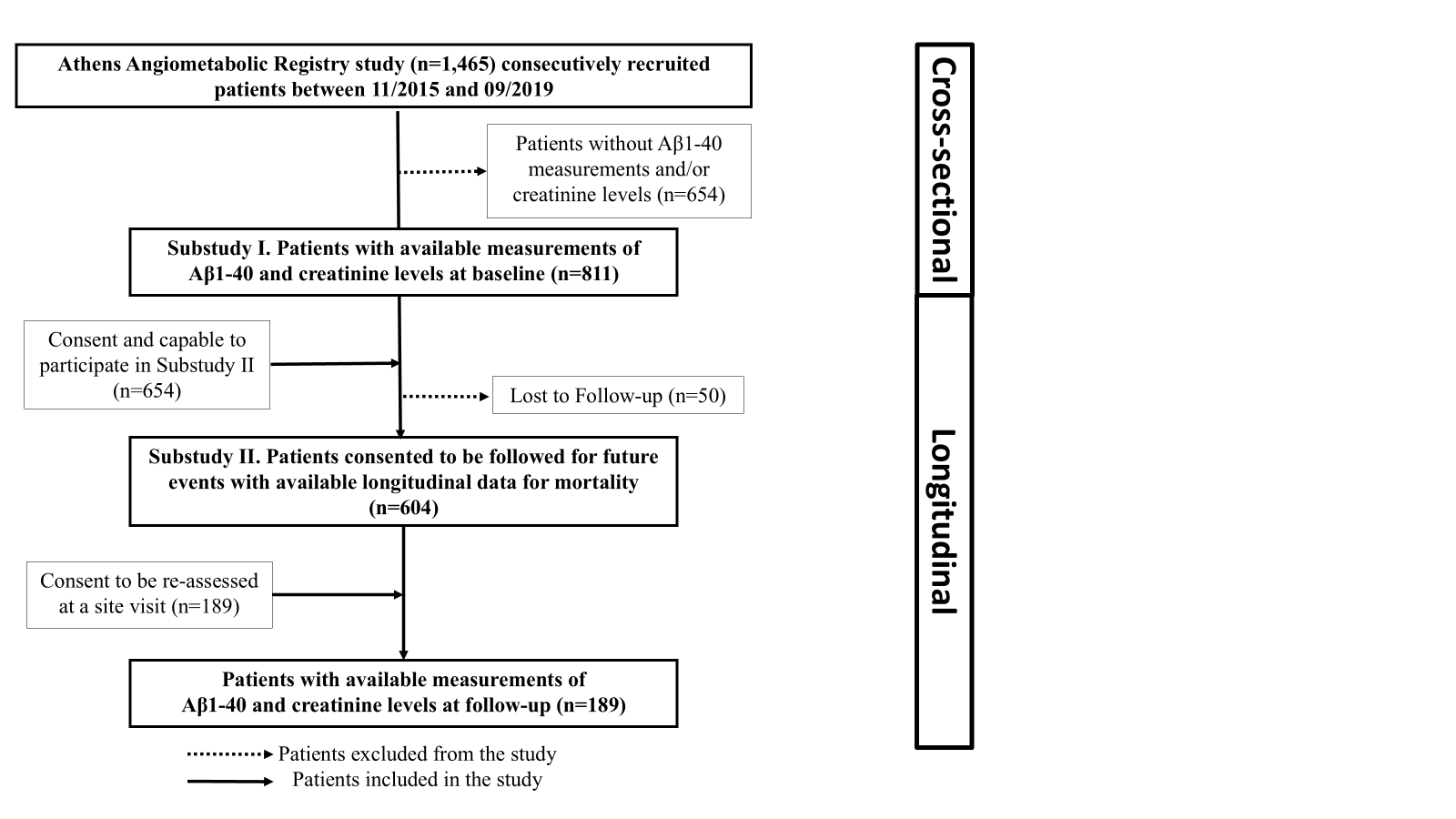


**Supplementary Figure 1. Flow chart of the study**

Abbreviations: Αβ1-40, amyloid-beta 1-40.
